# Osteoporosis Recovery by* Antrodia camphorata* Alcohol Extracts through Bone Regeneration in SAMP8 Mice

**DOI:** 10.1155/2016/2617868

**Published:** 2016-04-10

**Authors:** Hen-Yu Liu, Chiung-Fang Huang, Chun-Hao Li, Ching-Yu Tsai, Wei-Hong Chen, Hong-Jian Wei, Ming-Fu Wang, Yueh-Hsiung Kuo, Mei-Leng Cheong, Win-Ping Deng

**Affiliations:** ^1^Stem Cell Research Center, Taipei Medical University, Taipei 110, Taiwan; ^2^School of Dentistry, College of Oral Medicine, Taipei Medical University, Taipei 110, Taiwan; ^3^Department of Dentistry, Taipei Medical University Hospital, Taipei 110, Taiwan; ^4^Graduate Institute of Biomedical Materials and Tissue Engineering, Taipei Medical University, Taipei 110, Taiwan; ^5^Department of Food and Nutrition, Providence University, Taichung 433, Taiwan; ^6^Department of Chinese Pharmaceutical Sciences and Chinese Medicine Resources, China Medical University, Taichung 404, Taiwan; ^7^Department of Biotechnology, Asia University, Taichung 413, Taiwan; ^8^Department of Obstetrics and Gynecology, Cathay General Hospital, Taipei 106, Taiwan; ^9^College of Medicine, Taipei Medical University, Taipei 110, Taiwan; ^10^Institute of Medicine, Fu Jen Catholic University, Taipei 242, Taiwan

## Abstract

*Antrodia camphorata* has previously demonstrated the efficacy in treating cancer and anti-inflammation. In this study, we are the first to evaluate* Antrodia camphorata* alcohol extract (ACAE) for osteoporosis recovery* in vitro* with preosteoblast cells (MC3T3-E1) and* in vivo* with an osteoporosis mouse model established in our previous studies, ovariectomized senescence accelerated mice (OVX-SAMP8). Our results demonstrated that ACAE treatment was slightly cytotoxic to preosteoblast at 25 *μ*g/mL, by which the osteogenic gene expression (RUNX2, OPN, and OCN) was significantly upregulated with an increased ratio of OPG to RANKL, indicating maintenance of the bone matrix through inhibition of osteoclastic pathway. Additionally, evaluation by Alizarin Red S staining showed increased mineralization in ACAE-treated preosteoblasts. For* in vivo* study, our results indicated that ACAE inhibits bone loss and significantly increases percentage bone volume, trabecular bone number, and bone mineral density in OVX-SAMP8 mice treated with ACAE. Collectively,* in vitro* and* in vivo* results showed that ACAE could promote osteogenesis and prevent bone loss and should be considered an evidence-based complementary and alternative medicine for osteoporosis therapy through the maintenance of bone health.

## 1. Introduction

Osteoporosis is the most common bone disease and is characterized by low bone mass, microarchitectural deterioration of bone tissue, and subsequent bone fragility with susceptibility to fracture [1]. Bone fracture risk typically increases in the hip, vertebral, and distal forearm bones. These fractures are not only painful but also disabling, leading to the need for nursing home care and increased mortality when compared to age matched populations [2, 3]. Because of the high morbidity and mortality associated with osteoporotic fractures, treatment of osteoporosis prioritizes fracture prevention [4].

Multiple treatment options are currently available to osteoporosis patients including bisphosphonates, cell therapy, and supplementation of calcium and/or vitamin D; however, significant shortcomings and the continued widespread impact of the disease warrants further investigation into alternative treatments [5, 6]. Bisphosphonates effectively prevent bone loss through inhibition of osteoclastic bone resorption, but this tactic is one sided in that it does not affect bone renewal and has several adverse reactions ranging from mild to severe [7–9]. Cell therapy is a promising possibility but has many intrinsic hurdles to overcome such as the lack of bone homing ability in mesenchymal stem cells and the uncertainty of cell fate after implantation [10–12]. Vitamin D and calcium are components of bone renewal, but supplementation has limited and inconsistent effectiveness and is often used in combination with other treatments [13].

*Antrodia camphorata* (AC) is a traditional herbal medicine that is safe and contains osteogenic precursors that make it a likely candidate for effective osteoporosis therapy. AC is a* Ganoderma*-like fungus of the Polyporaceae Basidiomycota family composed of pharmacologically active components including steroids, triterpenoids, polysaccharides, lignans, phenyl derivatives, fatty acids, and trace elements [14, 15]. Much characterization and evaluation of AC components such as crude extracts, bioactivities, and pure compounds have already been completed [16]. Traditional medicines currently made from the fungus are used to treat digestion, hypertension, and pain along with exhibiting anti-inflammatory, antioxidative, and anticancer effects [17]. Recent studies on AC have also shown promising ability to protect the liver from oxidative stress and tissue injuries [18–21]. Previously, we showed that AC alcohol extracts (ACAE) inhibited non-small-cell lung cancer cell growth by promoting cell cycle arrest and inducing caspase 3-mediated apoptosis [18]. There are currently no studies on how AC affects osteoblasts, but many of the above mentioned components are known individually as factors in bone metabolism [22, 23]. AC was obtained from an artificial culture community for this study and concentrated into an alcohol extract (ACAE) to evaluate its potential as a preventative treatment for osteoporosis.* In vivo*, our study utilizes senescence accelerated mouse prone 8 (SAMP8), which was established through phenotypic inbreeding from a common genetic pool of the AKR/J mouse strain that exhibits osteoporosis and can be enhanced by ovariectomy, as established in our previous study. Therefore, ovariectomized-SAMP8 mice (OVX-SAMP8) were an appropriate animal model for* in vivo* study of ACAE on bone [10]. We hypothesized that ACAE treatment is slightly cytotoxic and could induce osteogenesis in preosteoblast* in vitro* and in osteoporotic mice* in vivo*. Subsequently, ACAE treatment may be an effective and safe alternative osteoporosis therapy.

## 2. Materials and Methods

### 2.1. Experimental Animals and ACAE Treatment

The female SAMP8 mice experiment protocol was approved by the Institutional Animal Care and Use Committee of Taipei Medical University. All applicable institutional and/or national guidelines for the care and use of animals were followed. The mice were maintained in the animal room under maintained conditions of 25°C and 50% relative humidity. The ovariectomized-SAMP8 female mouse was established by our previous study as an osteoporotic mouse model [10]. Female SAMP8 mice were ovariectomized at 4 months after birth to induce osteoporosis for experiments with 6 animals per group. The operation was performed on a SHAM-operated group of SAMP8 female mice at 4 months of age excluding removal of the ovaries. For 4 months following the ovariectomy operation 450 mg/kg/day by oral gavage was administered to the ACAE group, while the control group received phosphate buffered solution (PBS).

### 2.2. Cell Culture

MC3T3-E1 preosteoblastic (ATCC CRL-2593) cells were cultured in alpha minimum essential media (*α*-MEM) (Gibco) supplemented with 10% fetal bovine serum (FBS) (Gibco) and 1% Penicillin-Streptomycin-Amphotericin B (PSA) in a 10 cm culture dish.

### 2.3. Preparation of ACAE

AC fruiting bodies were cultured artificially and provided by Well Shine Biotechnology Development Co. (Taipei, Taiwan.) Finely powdered AC was combined with 95% ethanol in a 1 : 20 (w/v) ratio and shaken for 24 h at room temperature. The supernatant was extracted and filtered at a pore size of 0.2 mm (Millex GP Carrigtwohill, Cork, Ireland) and then centrifuged at 3000 rpm for 30 min to remove the precipitate. The extracts were lyophilized and stored at −20°C before use.

### 2.4. MTT Assay

MTT 3-(4,5-dimethylthiazol-2-yl)-2,5-diphenyltetrazolium bromide assay with tetrazolium salt reagent (Roche) was performed to determine the cytotoxicity of ACAE and EtOH on MC3T3-E1 preosteoblasts (EtOH data not shown). MC3T3-E1 cells were seeded into a 96-well plate at a density of 2 × 10^3^ cells/well and, after 24 hours, the media were changed to different concentrations of ACAE in media along with a control group that was cultured in *α*-MEM complete culture media only. MTT reagent was added into each well 24 hours after the treatment media. Four hours after the addition of MTT, the reagent was replaced with DMSO, and the optical density values were analyzed using Multiskan PC (Thermo Lab), and cell survival curves were plotted.

### 2.5. Alizarin Red S Staining for Osteogenesis

Osteogenesis was verified using Alizarin Red S staining. Cells were fixed with 10% formaldehyde (Merk) followed by 2% Alizarin Red S (pH 4.2) (Sigma) staining for 15 min at room temperature. For quantification, the bound staining was eluted with 10% cetylpyridinium chloride, and the absorbance of supernatants was measured at 540 nm [24].

### 2.6. RT-PCR Analysis

Total RNA was isolated from the test groups of MC3T3-E1 cells cultured in different concentrations of ACAE media using Trizol reagent (Invitrogen). Gene expression levels were measured by RT-PCR. Primer sequences were indicated as follows: Osteocalcin (OCN) forward primer 5′-CAGCTTGGTGCACACCTAAGC-3′; reverse primer 5′-AGGGTTAAGCTCACACTGCTCC-3′; temperature 55°C; Osteopontin- (OPN-) forward primer 5′-ATGA-GATTGGCAGTGATT-3′; reverse primer 5′-GTTGACCTCAGAAGATGA-3′; temperature 48.8°C; Runt-related transcription factor 2 (RUNX2) forward primer 5′-ACTTTCTCCAGGAAGACTGC-3′; reverse primer 5′-GCTGTTGTTGCTGTTGCTGT-3′; temperature 55°C; Receptor Activator of Nuclear Factor Kappa B (RANK) forward primer 5′-TCCAGGTCACTCCTCCATGC-3′; reverse primer 5′-GTTCCAGTGGTAGCCAGCCG-3′; temperature 66°C; glyceraldehyde 3-phosphate dehydrogenase (GAPDH) which was used as an internal control (CTRL) forward primer 5′-GCTCTCCAGAACATCATCCCTGCC-3′; reverse primer 5′-CGTTGTCATACCAGGAAATGAGCTT-3′; temperature 55°C. PCR products were separated by electrophoresis on 1% agarose gels (Agarose I; AMRESCO) and visualized with DNA View (Biotools, Taipei, Taiwan) staining.

### 2.7. Bone Imaging

Dual-energy X-ray absorptiometry (DEXA) (XR-36; Norland Corp.; host software revision 2.5.3, scanner software revision 2.0.0) analysis was used to establish measurements of bone mineral density in the spine and femur after 4 months of treatment. Bone samples from all groups were collected and imaged using a SkyScan-1076 MicroCT System (Skyscan, Belgium). The following three-dimensional (3D) parameters were measured: bone volume, total volume, and trabecular bone numbers. For trabecular bone analysis and 3D image construction, a MicroCT scanner (Skyscan-1076, Skyscan, Belgium) was operated at 50 kV, 200 *μ*A, 0.4 *μ* of rotation step, 0.5 mm Al filter, and 9 *μ*m/pixel of scan resolution. The data collected was quantitatively represented as the percentage of bone volume/total volume and the trabecular bone number (1/mm) [25].

### 2.8. Statistical Analysis

All results were represented as mean ± standard deviation (SD). Significant differences between two groups were determined by Student's *t*-test, *P* value <0.05. Figures were graphed using Sigma Plot 10.0.

## 3. Results

### 3.1. Dose Dependent Cytotoxicity of ACAE on Preosteoblasts

**Figure 1:**
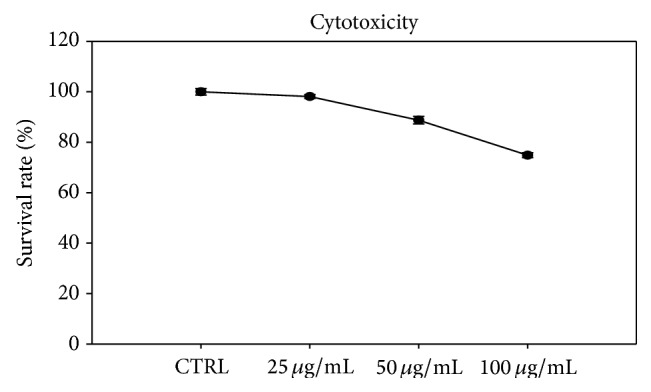
Cytotoxicity of ACAE on preosteoblasts. MTT assay was performed on preosteoblast cells after 24 h treatment with different concentrations of ACAE. Results are presented as percentages of cell viability. Representative results of 3 experiments demonstrated mean ± SD.

To determine the cytotoxicity of ACAE, the preosteoblasts MC3T3-E1 were exposed to ACAE at concentrations of 0, 25, 50, and 100 *μ*g/mL. The results indicated slight cytotoxic effect of ACAE on preosteoblast viability at 25 *μ*g/mL of ACAE: a 10% decrease in survival rate at 50 *μ*g/mL and a 13% decrease in survival rate at 100 *μ*g/mL (Figure 1). Therefore, ACAE at 25 *μ*g/mL was used as the experimental dosage for the subsequent study.

### 3.2. Osteogenesis of Preosteoblasts Treated with ACAE

**Figure 2:**
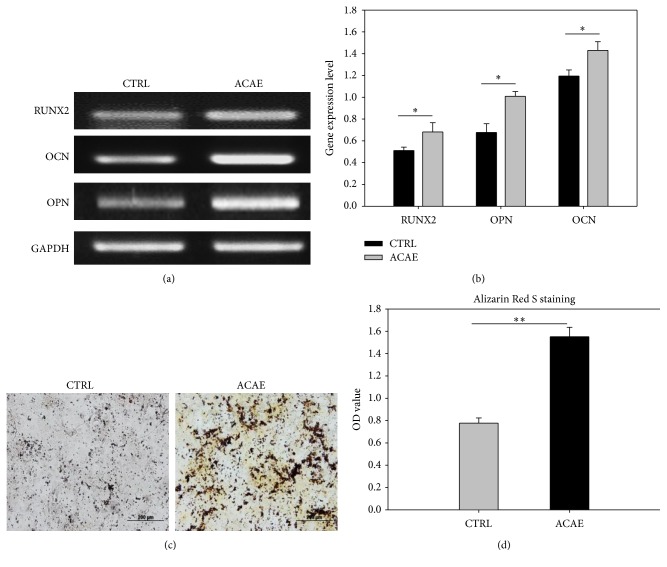
Analysis of* in vitro* osteoblastic differentiation of preosteoblasts treated with ACAE. (a) RT-PCR indicated the expression of osteogenic markers RUNX2, OCN, and OPN. (b) Quantitative analysis of the PCR results. (c) Alizarin Red S staining of preosteoblast mineralization. (d) Quantitative analysis of staining results. Representative results of 3 experiments demonstrated mean ± SD. ^*∗*^ *P* < 0.05; ^*∗∗*^ *P* < 0.01.

To further examine the degree of osteogenic differentiation in preosteoblasts in the presence of ACAE, Alizarin Red S staining and RT-PCR were performed. PCR was used to detect the degree of gene expression for the osteogenic markers: RUNX2, OCN, and OPN from a culture of preosteoblasts in *α*-MEM with 25 *μ*g/mL ACAE to compare to a control culture without ACAE. Stronger expression of all 3 osteogenic markers when cultured with ACAE was observed (Figure 2(a)). The quantitative analysis of the PCR results confirm that ACAE treatment of 25 *μ*g/mL resulted in significantly higher gene expression of RUNX2, OCN, and OPN, indicating increased osteogenic differentiation (Figure 2(b)). The results showed visibly darker and larger areas of Alizarin Red S staining in the ACAE-treated culture relative to the control, which indicates more mineralization of extracellular matrix (Figure 2(c)). Quantitative analysis confirmed significantly higher staining in the preosteoblasts in the ACAE culture than in the control (Figure 2(d)). This supports our PCR data that ACAE promotes osteogenic differentiation in preosteoblasts* in vitro*.

### 3.3. Analysis of OPG and RANKL Ratio in Preosteoblasts with ACAE

**Figure 3:**
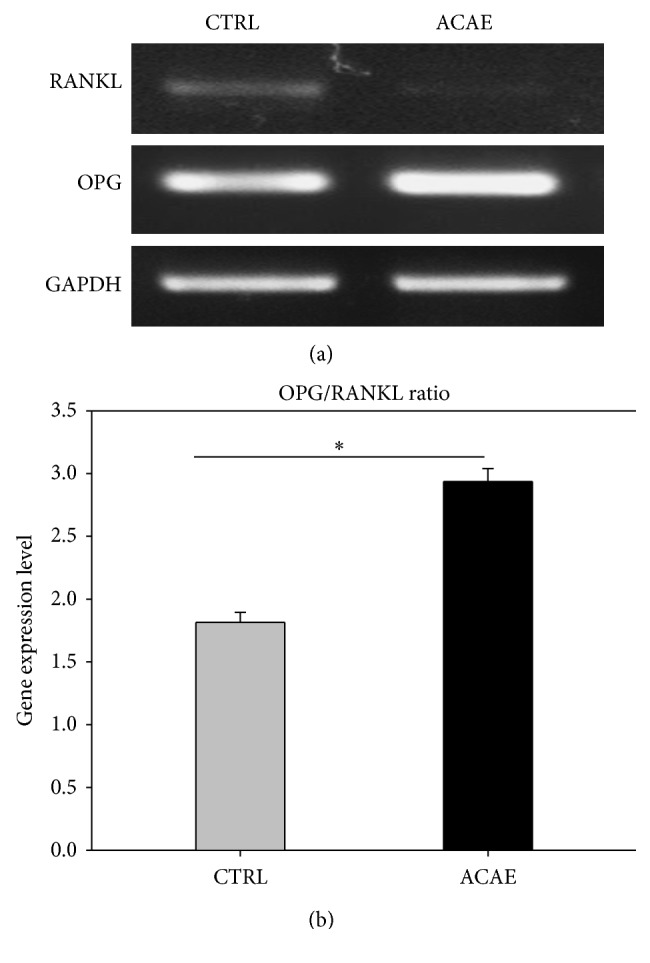
Analysis of osteogenic differentiation of preosteoblasts with ACAE. (a) RT-PCR indicated the expression of RANKL and OPG from a culture of preosteoblasts in *α*-MEM with 25 *μ*g/mL ACAE and control (CTRL) group. (b) Quantitative analysis of PCR results. Representative results of 3 experiments demonstrated mean ± SD. ^*∗*^ *P* < 0.05 versus CTRL group.

PCR was used to detect the degree of gene expression for the osteoclastogenic inhibitor OPG and RANKL which is essential to osteoclastogenesis, from a culture of preosteoblasts in *α*-MEM with 25 *μ*g/mL ACAE to the control group without ACAE. Stronger expression of OPG and weaker expression of RANKL were observed when cultured with ACAE (Figure 3(a)). The quantitative analysis of the PCR results confirms that ACAE treatment resulted in significantly higher gene expression ratio of OPG to RANKL (Figure 3(b)). Results indicate that ACAE promotes the maintenance of the bone matrix through upregulation of OPG and downregulation of RANKL.

### 3.4. Bone Mineral Density Increased with ACAE Treatment

**Figure 4:**
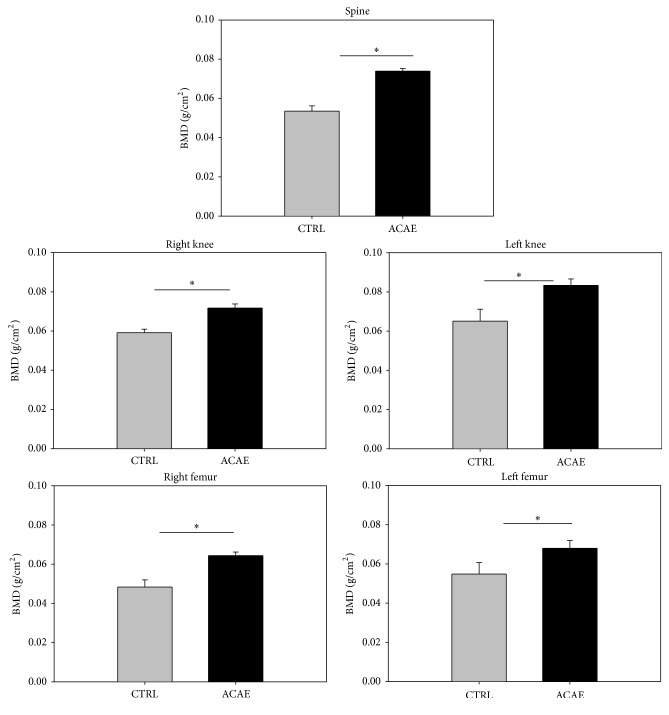
Bone mineral density with ACAE treatment. Bone mineral density (BMD) was measured by dual-energy X-ray absorptiometry for the spine, knees (right and left), and femurs (right and left). OVX-SAMP8 mice with ACAE treatment and CTRL group were measured at 4 months. Each bar represents the average from six animals. ^*∗*^ *P* < 0.05 versus CTRL group.

BMD was measured on mice after their ovariectomy at 0 months before the test group began ACAE treatment. After four months, BMD was measured on the SHAM-operated along with the OVX-operated CTRL and ACAE group. SHAM-operated mice showed a decrease in BMD, but the OVX-operated CTRL group had a significantly amplified loss of BMD (see supplementary Figure 1 in Supplementary Material available online at http://dx.doi.org/10.1155/2016/2617868). After four months of ACAE treatment, the CTRL and ACAE groups were measured for bone mineral density (BMD) at the spine, knees (right and left), and femurs (right and left) using dual-energy X-ray absorptiometry. The average BMD scores of the six mice were subjected to quantitative analysis revealing that ACAE treatment resulted in significantly higher BMD than the control group in all sites tested in the OVX-SAMP8 mice (Figure 4).

### 3.5. Analysis of Bone Quantity after ACAE Treatment

**Figure 5:**
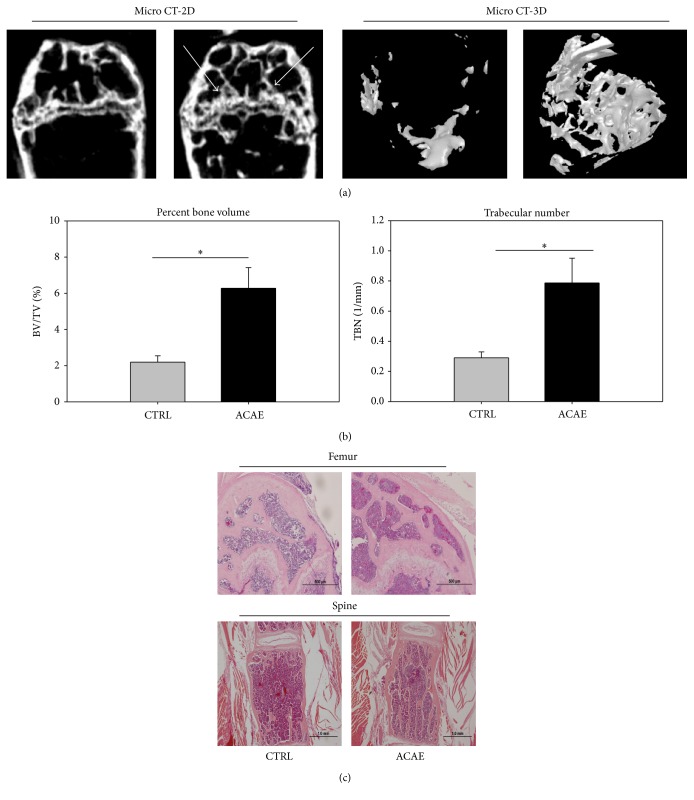
Analysis of bone quantity with photomicrographs. (a) 2D and 3D photomicrographs by MicroCT of femurs from OVX-SAMP8 mice with and without ACAE treatment. Arrows indicate areas of visibly higher bone volume in the MicroCT-2D. (b) Quantitative analysis of photomicrographs to determine percentage bone volume (PBV) and trabecular number (TBN). (c) Histological slides of the femur and spine with ACAE treatment and a control. Each bar represents the average from six animals. ^*∗*^ *P* < 0.05 versus CTRL group.

Photomicrographs using both 2D and 3D MicroCT displayed both higher percentage bone volume (PBV) and trabecular number (TBN) with ACAE. The arrows indicate areas of visibly higher bone volume in the MicroCT-2D (Figure 5(a)). Quantitative analysis of the photomicrographs showed a greatly increased PBV and TBN relative to the control group (Figure 5(b)). Histological slides showed higher ratios of trabecular bone in the femur and spine with ACAE relative to the control which showed more space (Figure 5(c)).

## 4. Discussion

The aim of this study was to determine the potential effects of* Antrodia camphorata* alcohol extracts (ACAE) in osteoporosis therapy. Our results suggest that ACAE could prevent bone loss and significantly induce bone recovery from osteoporosis by balancing bone remodeling. These findings provide the first reports of ACAE in bone regeneration and support it as a promising candidate for safe and effective osteoporosis therapy.

*Antrodia camphorata* (AC) has many previously explored medicinal properties in addition to newly explored potential in promoting osteoblast differentiation [18, 26]. In this study, we found that ACAE treatment of preosteoblasts (MC3T3-E1) was slightly toxic by MTT analysis while inducing osteoblastic differentiation. These findings support that not only is ACAE a promising cancer therapy but also it has the potential to promote osteogenesis in osteoporosis therapy. The potential of ACAE in osteogenesis was unknown and not yet investigated in previous studies; however, AC contains multiple components such as higher triterpenoids, polysaccharides (*β*-D Glucosan), ergosterol, and trace elements (calcium, phosphatase, germanium, and chitosan) [27], which are factors associated in the induction of osteogenic differentiation. Triterpenoids have been shown to exhibit significantly protective effects on bone remodeling regulation in osteoporosis therapy [28], while polysaccharides and polysaccharide-based scaffold promote osteogenesis [29], and ergosterol is a vitamin D precursor which is known to stimulate osteoblastic differentiation [30]. Furthermore, our RT-PCR analysis results demonstrated that ACAE treatment upregulated the gene expression of RUNX2, OPN, and OCN, along with strong mineralization of bone matrix observed by Alizarin Red S staining. Additionally, recent studies indicated that AC provides trace elements that contribute to bone health and showed that vitamin D and Ca deficiency increase the risk of osteoporosis and bone fractures [31, 32]. The above mentioned studies collectively support our finding that AC has the potential to induce osteogenesis in an osteoporotic animal model.

Current therapies widely depend on bisphosphonates to treat osteoporosis [33]. Although these drugs prevent bone loss and decrease the risk of bone fractures in osteoporosis patients, there are many adverse reactions including upset stomach, erosion of the esophagus, flu-like symptoms, osteonecrosis of the jaw, intense musculoskeletal pain, atrial fibrillation, and atypical femur fractures [7]. Additionally, bisphosphonates carry warnings and contraindications for patients with reduced renal function, in contrast to AC which has demonstrated hepatoprotective qualities [34, 35]. Finally, bisphosphonates only mediate bone resorption through osteoclast inhibition without promoting bone formation [36], while ACAE has the potential to induce osteogenesis and could therefore greatly improve on current osteoporosis therapy. Our results demonstrated that ACAE could promote osteoblast differentiation and suppress osteoclastic differentiation by inhibiting RANKL expression and strongly increasing OPG expression in ACAE-treated OVX-SAMP8 mice in addition to improving bone density which collectively ameliorated osteoporosis in OVX-SAMP8 mice. Interestingly, this result is supported by previous finding that triterpenoids could inhibit osteoclast formation by reducing RANKL expression [37], suggesting that the triterpenoids in ACAE may be useful compounds for modulating bone resorption in osteoporosis therapy.

ACAE is an herbal medicine extract and is therefore a cocktail containing a multitude of components. The cellular responses to each pure compound as well as the mechanisms involved in producing therapeutic results have not been fully explored. Research on traditional herbal drugs is an important means to finding new drugs; however, their true properties may remain unknown even after studies have demonstrated their effectiveness [38]. Further examination of ACAE is warranted through the evaluation of its pure compounds. However, the ACAE cocktail was able to modulate osteogenesis in osteoporotic mice, and our results demonstrated that ACAE prevented bone loss and significantly increased percentage bone volume, trabecular bone number, and bone mineral density in OVX-SAMP8 mice. These findings and those of previous studies may be a product of synergistic activity that could promote osteogenesis in osteoporosis therapy. Although the key factors regulating the observed functional recovery are not fully characterized, the treatment potential may differ in every pure compound when used alone. We found that* in vitro* and* in vivo* results show promotion of osteogenesis and the potential to prevent osteoporosis by treatment with ACAE.

In this study we demonstrated that ACAE has the potential to maintain bone health through promotion of osteogenesis and the inhibition of bone digestion. The progression of cancer is linked to osteoporosis, and Martin et al. found that healthy bone could suppress bone metastasis. Maintaining bone volume and function could therefore reduce tumor invasion in breast cancer patients [39–41]. Subsequently, our results indicate that AC could potentially reduce bone metastasis in patients suffering from breast cancer and osteoporosis along with strongly inhibiting cancer growth, as shown in our previous study [18]. Our study supports the use of AC as an evidence-based complementary and alternative medicine for cancer therapy and osteoporosis in addition to other associated bone diseases through maintenance of bone health.

## 5. Conclusion

This study indicated ACAE promotion of osteogenesis* in vitro* in MC3T3-E1 preosteoblasts and* in vivo* in the OVX-SAMP8 osteoporotic mouse model in addition to inhibited RANKL relative to OPG (osteoclastogenesis). Our results demonstrated significant bone recovery and decreased bone loss that indicate improved bone remodeling balance and that ACAE could be a uniquely well-balanced treatment for osteoporosis.

## Supplementary Material

Female SAMP 8 mice were ovariectomized at 4 months after birth to induce osteoporosis. The operation was performed on a SHAM-operated group of SAMP8 female mice at 4 months of age excluding removal of the ovaries. For 0 month non-induced ovaiectomy and after 4 months with or without ACAE treatment following the ovariectomy operation OVX-SAMP8 mice were measured the bone mineral density (BMD) by dual-energy X-ray absorptiometry analysis.
