# Corrigendum to “Osteoporosis Recovery by* Antrodia camphorata* Alcohol Extracts through Bone Regeneration in SAMP8 Mice”

**DOI:** 10.1155/2017/6297946

**Published:** 2017-02-15

**Authors:** Hen-Yu Liu, Chiung-Fang Huang, Chun-Hao Li, Ching-Yu Tsai, Wei-Hong Chen, Hong-Jian Wei, Ming-Fu Wang, Yueh-Hsiung Kuo, Mei-Leng Cheong, Win-Ping Deng

**Affiliations:** ^1^Stem Cell Research Center, Taipei Medical University, Taipei 110, Taiwan; ^2^School of Dentistry, College of Oral Medicine, Taipei Medical University, Taipei 110, Taiwan; ^3^Department of Dentistry, Taipei Medical University Hospital, Taipei 110, Taiwan; ^4^Graduate Institute of Biomedical Materials and Tissue Engineering, Taipei Medical University, Taipei 110, Taiwan; ^5^Department of Food and Nutrition, Providence University, Taichung 433, Taiwan; ^6^Department of Chinese Pharmaceutical Sciences and Chinese Medicine Resources, China Medical University, Taichung 404, Taiwan; ^7^Department of Biotechnology, Asia University, Taichung 413, Taiwan; ^8^Department of Obstetrics and Gynecology, Cathay General Hospital, Taipei 106, Taiwan; ^9^Department of Obstetrics and Gynecology, School of Medicine, College of Medicine, Taipei Medical University, Taipei 110, Taiwan; ^10^Institute of Medicine, Fu Jen Catholic University, Taipei 242, Taiwan

In the article titled “Osteoporosis Recovery by* Antrodia camphorata* Alcohol Extracts through Bone Regeneration in SAMP8 Mice” [1], affiliation number nine was incomplete. The corrected affiliation is shown above.
