# Supplementary figures and images for: Osteoporosis Recovery by Antrodia camphorata Alcohol Extracts through Bone Regeneration in SAMP8 Mice
Source: Evid Based Complement Alternat Med. 2016 Apr 10;2016:2617868. doi: 10.1155/2016/2617868 (PMC4842042; doi:10.1155/2016/2617868)

# B M D analysis

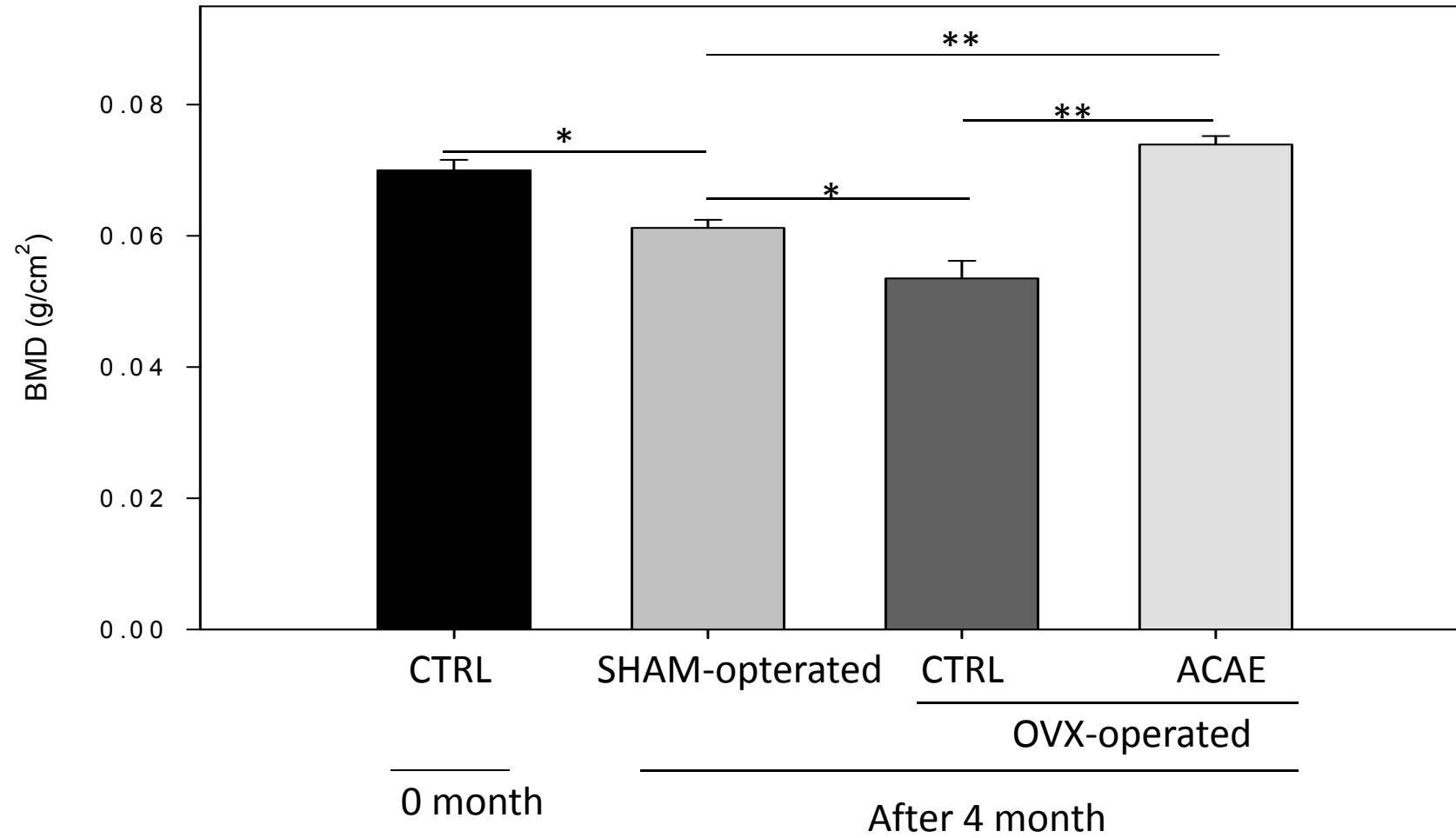

Supplement: Supplementary file 1 — Female SAMP 8 mice were ovariectomized at 4 months after birth to induce osteoporosis. The operation was performed on a SHAM-operated group of SAMP8 female mice at 4 months of age excluding removal of the ovaries. For 0 month non-induced ovaiectomy and after 4 months with or without ACAE treatment following the ovariectomy operation OVX-SAMP8 mice were measured the bone mineral density (BMD) by dual-energy X-ray absorptiometry analysis. [file 2617868.f1.pdf]
